# Antimicrobial Resistance, Virulence Factors, and Pathotypes of *Escherichia coli* Isolated from Drinking Water Sources in Jordan

**DOI:** 10.3390/pathogens8020086

**Published:** 2019-06-25

**Authors:** Samer Swedan, Heba Abu Alrub

**Affiliations:** Department of Medical Laboratory Sciences, Jordan University of Science and Technology, Irbid 22110, Jordan; htabualrub13@ams.just.edu.jo

**Keywords:** *Escherichia coli*, pathotype, resistance, antibiotic, beta lactamase, carbapenemase, plasmid

## Abstract

The study investigated the prevalence of potentially pathogenic and drug resistant *Escherichia coli* among drinking water sources in Jordan. A total of 109 confirmed *E. coli* isolates were analyzed. Antimicrobial susceptibility testing was done using the Kirby Bauer disk diffusion method. Phenotypic identification of extended spectrum beta-lactamase (ESBL) and carbapenemase production was done using the double disk synergy test and the modified Hodge test, respectively. Isolates’ plasmid profiles were determined by gel electrophoresis. PCR was used for detection of virulence and resistance genes. Overall, 22.0% of the isolates were potentially intestinal pathogenic *E. coli* (IPEC); namely enteroaggregative *E. coli* (16.5%), enteropathogenic *E. coli* (2.8%), enteroinvasive *E. coli* (1.8%), and enterohemorrhagic *E. coli* (0.9%). A third of the isolates were multi-drug resistant. The highest rates of antimicrobials resistance were observed against ampicillin (93.6%) and sulfamethoxazole/trimethoprim (41.3%). All isolates were susceptible to imipenem, meropenem, doripenem and tigecycline. The prevalence of ESBL and carbapenemase producers was 54.1% and 2.8%, respectively. *Bla_VIM_* was the most prevalent resistance gene (68.8%), followed by *bla_CTX_* (50.5%), *bla_TEM_* (45.9%), *bla_NDM_* (11%), *bla_KPC_* (4.6%), and *bla_SHV_* (0.9%). Fifty-eight (53.2%) isolates contained one or more plasmid ranging from 1.0 to 8.0 kbp. Overall, high prevalence of potentially pathogenic and resistant isolates was observed.

## 1. Introduction

Diarrheal diseases are considered major infectious diseases leading to high rates of morbidity and mortality worldwide [1]. Diarrheal diseases occur more commonly in developing countries and particularly among children, in which diarrhea is considered the second-most common cause (after pneumonia) of death under the age of five [2]. The lack of safe water supplies, use of contaminated water sources, inadequate sanitation, and poor hygiene are the main risk factors for acquiring diarrhea in developing countries [2].

Most of the known *E. coli* strains are members of the gut normal flora. However, some strains are considered true pathogens, capable of causing urinary tract infections, sepsis, meningitis, and enteric or diarrheal diseases [3]. *E. coli* is an etiologic agent of diarrhea in developing countries. It is responsible for major waterborne bacterial infections and is successfully transmitted through the direct intake of contaminated water or indirectly through food crops exposed to contaminated water sources [3]. Diarrheagenic *E. coli* strains also known as intestinal pathogenic *E. coli* (IPEC) have been grouped into six pathotypes: Enteropathogenic *E. coli* (EPEC), enterotoxigenic *E. coli* (ETEC), enterohemorrhagic *E. coli* (EHEC), enteroinvasive *E. coli* (EIEC), enteroaggregative *E. coli* (EAEC), and diffusely adherent *E. coli* (DAEC) [4]. These strains contain virulence genes which can be used for strain identification. The virulence genes are encoded on mobile genetic elements which enable their spread to other bacteria [5].

The attaching and effacing gene (*eae*) is found in EPEC and EHEC. This gene is associated with the development of attaching and effacing lesions in the intestinal epithelium [5,6]. The EAEC which usually causes persistent diarrhea can be identified by detection of the AggR-activated island (*aaiC*) gene [5,7]. The EHEC causes severe diseases such as bloody diarrhea and hemolytic uremic syndrome due to the release of shiga toxin [8]. The production of shiga toxins encoded on *stx1* and/or *stx2* genes is what distinguishes EHEC from other *E. coli* pathotypes, such as EPEC [5]. The EIEC is similar to *Shigella* spp. in multiple aspects such as the invasion of the colon, and is usually characterized by the presence of the *ipaH* gene [3,5].

The recent rise in antimicrobial resistance rates among *E. coli* is significantly contributing to treatment difficulties and failures leading to increased morbidity and mortality among patients [9]. Some of the mechanisms of resistance include the production of extended-spectrum lactamases (ESBLs) and carbapenemases. ESBLs are plasmid-mediated enzymes having the ability to hydrolyze beta-lactam antimicrobial agents including penicillins, cephalosporins, and aztreonam. ESBLs can be classified into three main types; TEM, SHV, and CTX-M [10,11]. Carbapenemases are a major group of beta-lactamases capable of hydrolyzing penicillins, cephalosporins, monobactams, and carbapenems. They include beta-lactamases of classes B (e.g., IMP and VIM), D (e.g., OXA-23 to -27), and A (e.g., IMI, KPC, NMC, and SME) [12,13].

The contamination of the water supply with pathogenic *E. coli* strains is a serious risk factor for spreading waterborne infections in humans [14]. On the other hand, the presence of resistant *E. coli* strains in the water supply and the emergence of antimicrobial resistance mechanisms, especially those associated with mobile genetic elements, may enhance the possibility of simultaneous spreading of antimicrobial resistance and virulence genes to other bacteria, leading to the emergence of resistant pathogens [15].

Knowledge about the prevalence of pathogenic *E. coli* strains among sources of the drinking water supply in Jordan and in many developing countries is lacking. In this study, we identified the prevalence of potentially IPEC among drinking water sources in Jordan, antimicrobial susceptibility genotypes and phenotypes, and bacterial plasmid profiles. Overall, 22.0% of the *E. coli* isolates were potentially pathogenic strains. Multi-drug resistance, ESBL production, and carbapenemase production, were observed among the isolates at different rates. This and other similar studies [4,16,17,18,19], enable policy makers to undertake necessary actions to improve water safety via monitoring the occurrence of serious pathogens and drug resistance. 

## 2. Results

### 2.1. Identification of E. coli Pathotypes 

Among 109 confirmed *E. coli* isolates recovered from drinking water sources, 24 (22.0%) were potentially IPEC, as determined by the presence of characteristic virulence genes. The majority of the potentially pathogenic isolates (16.5%; 18/109) were EAEC which were positive for either *aat* (12.8%; 14/109) or *aaic* (2.8%; 3/109), or both genes (0.9%; 1/109), followed by EPEC which were positive for *eae* and negative for *stx1* and *stx2* (2.8%; 3/109), EIEC which were positive for *ipaH* (1.8%; 2/109), and one isolate was putatively considered EHEC as it was positive for *stx1* but surprisingly negative for *eae* (0.9%; 1/109). None of the isolates were positive for *stx2*. The remaining isolates (78.0%; 85/109) lacked any of the investigated virulence genes.

### 2.2. Antimicrobial Susceptibility

The antimicrobial susceptibility profile of the isolates to various antimicrobial agents is shown in Table 1. The highest rates of resistance were observed against ampicillin (93.6%), followed by sulfamethoxazole/trimethoprim (41.3%), ciprofloxacin (16.5%), levofloxacin (14.7%), ceftriaxone/cefotaxime (12.9%) and ceftizoxime (9.2%). All isolates were sensitive to imipenem, meropenem, doripenem and tigecycline. Resistance to three or more of the antimicrobial agent groups i.e., multi-drug resistance (MDR) phenotype, was seen among 34.9% of the isolates (Table 2).

Based on the results of the five antimicrobial agents used in the double disk synergy test (DDST) to detect ESBL producers, 59 ESBL producers were identified. Augmentation of the inhibition zone which indicates an ESBL producer was observed most frequently with ceftizoxime and cefotaxime (79.7% each; 47/59), followed by aztreonam (72.9%; 43/59), ceftriaxone (67.8%; 40/59), and ceftazidime (49.2%; 29/59). The correlation between the ESBL phenotype and susceptibility to antimicrobial agents is shown in Appendix A. A significant association was observed between the ESBL phenotype and nonsusceptibility to amoxicillin/clavulanic acid, aztreonam, cefotaxime, ceftazidime, ceftizoxime, and ceftriaxone. 

Based on the results of the four carbapenem drugs used in the modified Hodge test (MHT), three carbapenemase producing isolates were identified. The MHT positive isolates gave positive results only with ertapenem and imipenem (66.7% each; 2/3). The correlation between the carbapenemase phenotype and susceptibility to antimicrobial agents is shown in Appendix B. A significant association was observed between the carbapenemase phenotype and nonsusceptibility to amoxicillin/clavulanic acid (100%; 3/3), aztreonam (66.7%; 2/3), cefotaxime (66.7%; 2/3), ceftazidime (66.7%; 2/3), ceftizoxime (66.7%; 2/3), and ceftriaxone (66.7%; 2/3). No significant association was observed between the carbapenemase and ESBL phenotypes (Table 3).

### 2.3. Beta-Lactamase Resistance Genes

The beta-lactamase genes were detected among the isolates at the following rates: *bla_VIM_* (68.8%; 75/109), *bla_CTX_* (50.5%; 55/109), *bla_TEM_* (45.9%; 50/109), *bla_NDM_* (11%; 12/109), *bla_KPC_* (4.6%; 5/109), and *bla_SHV_* (0.9%; 1/109). Co-associations were observed between *bla_KPC_* and *bla_TEM_* (*p* = 0.018), while gene independence was observed between *bla_NDM_* and *bla_VIM_* (*p* = 0.008) (Table 4). There were significant associations between *stx1* with *bla_KPC_*, *aaic* with *bla_TEM_*, and *aat* with *bla_VIM_* (Table 5).

### 2.4. Plasmid Profiling

Among the 109 isolates, 58 (53.2%) had one or more plasmid (Table 6). Plasmid size estimates ranged from 1.0 to 8.0 kbp. The plasmid profile of each of the isolates was distinct as none of the isolates shared similar numbers or sizes of plasmids (Appendix C). No significant associations were observed between number of plasmids and number of resistance genes (Table 6), or between number of plasmids and the resistance to antimicrobial agent groups (Table 7).

## 3. Discussion

*E. coli* is a biological indicator of fecal contamination of water sources. It is a well-known potential pathogen responsible for a variety of waterborne infections in humans, such as gastrointestinal illness. Furthermore, the presence of antimicrobial resistant pathogenic *E. coli* strains in water sources may contribute to the spreading of antimicrobial resistance and virulence genes among other bacteria in the environment. In this study, *E. coli* was recovered from various drinking water sources in Jordan. All isolates were subjected to PCR detection of virulence genes associated with *E. coli* intestinal pathotypes, plasmid profiling, and phenotypic and genotypic analysis of antimicrobial resistance.

Using PCR to detect virulence genes, 22.0% of the isolates were identified as potentially IPEC. Hence, *E. coli* obtained from water sources can potentially lead to serious disease. The most prevalent potential pathotype was EAEC accounting for 16.5% of the isolates, followed by EPEC at 2.8%, EIEC at 1.8% and putatively EHEC at 0.9%. The one isolate identified putatively as EHEC, was not positive for *eae*, as is typical for this pathotype. This may be attributed to mutations in the gene preventing PCR amplification. Alternatively, this could be a non-EHEC strain that acquired *stx1* via horizontal gene transfer. Isolates demonstrated highest resistance rates against ampicillin and sulfamethoxazole/trimethoprim. All isolates were sensitive to imipenem, meropenem, doripenem and tigecycline. Many agents also demonstrated high susceptibility. Nonetheless, approximately one third of the isolates had an MDR phenotype.

According to the DDST, 54.1% of the isolates were ESBL producers. Various types of antimicrobials were used for the phenotypic detection of ESBL producers to enhance detection sensitivity. Ceftizoxime and cefotaxime identified the highest number of ESBL isolates, while ceftazidime identified the least. The CLSI (2015) recommends using multiple agents, including aztreonam, cefotaxime and ceftizoxime for ESBL screening, which is consistent with our methodology and findings. All ESBL positive isolates were susceptible to nitrofurantoin, tigecycline, imipenem, meropenem, and doripenem. Similar results have been reported for *E. coli* in Iran, South Africa, and Bangladesh [16,19,20,21]. The observed high rates of MDR are likely due to the dissemination of resistance genes via horizontal gene transfer among bacteria and the relative ease for *E. coli* to acquire genetic material, especially in light of increased selective pressure due to exposure to a wide range of antimicrobials in the human body, as well as in the environment.

A study from Jordan in 2012 on clinical *E. coli* isolates reported an ESBL rate of 50.3%; (83/165) [22]. A rate surprisingly similar to what we found, suggesting that *E. coli* obtained from the drinking water sources are potentially a subset of those involved in clinical infections. For comparison, 55.6% of clinical *E. coli* isolates from India were ESBL positive [23], while lower rates of ESBL producing clinical *E. coli* were reported in Qatar (34.7%), Iran (21%), Oman (13.3%), and Korea (9.2%) [24,25,26,27]. The higher prevalence of ESBL isolates in Jordan compared to other countries may be attributed to the trend of self-medication, unregulated prescription of antimicrobial agents, and the extensive prophylactic misuse of antimicrobials by Jordanian patients and physicians.

While the CLSI (2015) only recommends using ertapenem and meropenem for carbapenemase screening, this study utilized four carbapenem drugs for screening of carbapenemases using the MHT. Based on this, three (2.8%) isolates were identified as carbapenemase producers. Carbapenemase positive isolates were only identified using ertapenem and imipenem. Surprisingly, these isolates were still susceptible to all four carbapenem drugs. This could be attributed to the production of low carbapenemase levels, or a false positive MHT mostly generated by ESBL production in association with decreased drug permeability [28]. For comparison, in Lebanon the reported prevalence rate of carbapenemase production was 2.2% among clinical *E. coli* isolates [29]. MDR *E. coli* harboring ESBLs and carbapenemases in water sources is alarming, especially considering that carbapenems are usually the antimicrobials of last resort to treat ESBL-producing pathogens.

Different beta-lactamase genes were detected among the isolates; namely, *bla_VIM_* (68.8%) followed by *bla_CTX_* (50.5%), *bla_TEM_* (45.9%), *bla_NDM_* (11%), *bla_KPC_* (4.6%), and *bla_SHV_* (0.9%). *Bla_VIM_* was the most prevalent gene. This may be due to its presence on highly mobile genetic elements that facilitate its spread among bacteria and that it is the most common ESBL gene distributed worldwide [30]. The presence of *bla_VIM_* in Jordan was documented previously in clinical isolates [31].

The presence of ESBL-producing *E. coli* possessing *bla*_CTX_ genes in the environment was documented in different parts of the world [17,32,33]. Salah et al. reported a very high rate of 94.2% of *E. coli* producing CTX-M-type ESBLs in the intestines of Jordanian infants [34]. The high rate of *E. coli* harboring CTX-M-type ESBLs reported here is consistent with its high prevalence among the intestinal flora reported by Salah et al. and may be attributed to improper clinical waste management leading to contamination of water sources. Consistent with the findings of our study, 50% of the isolates from water sources in Bangladesh harbored *bla*_CTX_ [21]. The frequency of *bla_TEM_* among the isolates was similar to those reported from Qatar and Malaysia among water isolates [18,24].

Significant associations were observed between *stx1* and *bla_KPC_*, *aaic* and *bla_TEM_*, and *aat* with *bla_VIM_*. However, only one *stx1* positive isolate carried *bla_KPC_*. Therefore, this association may not be necessarily typical. The co-presence of virulence genes and resistant genes in pathogenic strains likely favors their survival and persistence in the host and the environment [35]. Similarly, the co-presence of *bla_CTX_* and *stx1* was described in *E. coli* isolates from Japan and France [36,37]. A study from Iran also reported the presence of *bla_CTX_* and *bla_TEM_* in EAEC [38].

Significant co-association was observed between *bla_KPC_* and *bla_TEM_*, which may reflect co-existence of the genes, their dissemination on mobile genetic elements via horizontal gene transfer, and the ability of bacteria to acquire several resistance genes. Similarly, the co-association of *bla_KPC_* and bla*_TEM_* was reported in *E. coli* clinical isolates from Taiwan and Greece [39,40].

In the present study, about half of the isolates carried one or more plasmid. This was similar to findings of a study from India in which 52.6% of *E. coli* isolates carried plasmids [41]. Plasmid size estimates ranged from 1.0 to 8.0 kbp. These findings are in agreement with studies from Bangladesh and Pakistan [42,43].

No significant associations were observed between the number of plasmids and number of resistance genes, suggesting that resistance genes might have translocated from plasmids to the chromosome, and/or that they were carried on a limited number of plasmids. Furthermore, there were no significant associations between resistance to various antimicrobial agent groups and number of plasmids. This may be due to the presence of genes encoding antibiotics resistance on plasmids as well as the bacterial chromosome. Similarly, some strains containing more than one plasmid were reported to be only resistant to one antimicrobial agent, and vice versa [44]. Other studies also reported no correlation between the number of plasmids and resistance patterns [45].

Due to the identification of potentially IPEC having high rates of drug resistance among drinking water sources, we suggest implementation of wider-scale studies to characterize their presence and the presence of other potential pathogens, and the identification of isolates’ virulence and antimicrobial resistance determinants. Furthermore, periodical monitoring of *E. coli* and other pathogens among water sources is required to assess their contribution to waterborne infections and health of the population, and to evaluate if water sources may serve as reservoirs for these pathogens. 

## 4. Materials and Methods

This study was approved by the Jordan University of Science and Technology (JUST) research committee. Requirement for approval by the institutional review board of JUST was waived as the study did not involve the study of human subjects, human data or tissue, or animals.

### 4.1. Bacterial Isolates

A total of 157 suspected *Escherichia coli* isolates were obtained from the water testing authority’s microbiology laboratory, Amman, Jordan from February to June 2015. Water samples processed at this laboratory originated from all sources of the drinking water supply in Jordan. Isolates were obtained from fluorescent wells of a Colilert Quanti-Tray (IDEXX Laboratories, Westbrook, ME, USA) and streaked onto MacConkey agar, followed by subculture on eosin-methylene blue agar. The identity of all isolates was confirmed using conventional methods, and PCR via detection of the *E. coli*-specific gene *uspA*. Of the total 157 isolates, 109 were confirmed as *E. coli*. For long term storage, overnight colonies from agar media were suspended in LB broth supplemented with glycerol (16% final concentration), and stored at −80 °C. Bacterial colonies from overnight cultures on Muller Hinton agar (MHA) were resuspended in 2.0 mL of sterile normal saline, to create a bacterial suspension having a turbidity equivalent to 0.5 McFarland for antimicrobial susceptibility testing (AST).

### 4.2. Antimicrobial Susceptibility Testing

AST was performed using the Kirby–Bauer disk diffusion method according to the Clinical and Laboratory Standards Institute (CLSI, 2015) guidelines. Any isolate showing a resistant or intermediately susceptible result using the Kirby–Bauer disk diffusion method was considered nonsusceptible to the respective antimicrobial agent. The antimicrobial disks used were amoxicillin/clavulanic acid (30 μg), aztreonam (30 μg), cefotaxime (30 μg), ceftazidime (30 μg), ceftizoxime (30 μg), ceftriaxone (30 μg), ampicillin (10 μg), ciprofloxacin (5 μg), levofloxacin (5 μg), nitrofurantoin (300 μg), sulfamethoxazole-trimethoprim (25 μg), tigecycline (15 μg), doripenem (10 μg), ertapenem (10 μg), imipenem (10 μg), and meropenem (10 μg). All disks were obtained from Oxoid (Basingstoke, Hampshire UK). *E. coli* clinical isolates having known AST profiles as determined by VITEK (bioMérieux, Marcy-l’Étoile, France) were used as positive and negative controls.

### 4.3. Detection of ESBLs

The production of ESBLs was detected by the double disk synergy test (DDST) according to the CLSI (2015) guidelines using a disk of amoxicillin/clavulanic acid along with aztreonam, cefotaxime, ceftazidime, ceftizoxime, and ceftriaxone. An MHA plate was inoculated with each isolate. Next, an amoxicillin/clavulanic acid disk was placed in the center of the plate, and aztreonam, cefotaxime, ceftazidime, ceftizoxime, ceftriaxone disks were placed 25 mm (center to center) from the amoxicillin/clavulanic acid disk. After overnight incubation at 37 °C, any distortion or increase in the zone of inhibition (i.e., augmentation of inhibition) towards the amoxicillin/clavulanic acid disk was considered a positive result for ESBL production. *Klebsiella pneumoniae* ATCC 700603 was used as positive control. *E. coli* ATCC 25922 was used as negative control.

### 4.4. Detection of Carbapenemase Production

Carbapenemase production was evaluated using the Modified Hodge test (MHT) according to the CLSI (2015) guidelines. Briefly, a 0.5 McFarland-equivalent suspension of *E. coli* ATCC 25922 in 5 mL of sterile saline was prepared. A 1:10 dilution was prepared in sterile saline. The diluted suspension was used to inoculate the full surface of an MHA plate. The plate was dried for 5 min and a disk of either doripenem (10 μg), ertapenem (10 μg), imipenem (10 μg), or meropenem (10 μg), was placed in the center of the plate. Two-to-four colonies of the test organism were selected and streaked in a straight line, from the edge of the disk, up to the edge of the plate. After overnight incubation at 36 °C, carbapenemase production was identified by observing a clover leaf-like indentation of *E. coli* ATCC 25922 growing along the test organism’s growth streak within the disk diffusion zone [46]. *K. pneumoniae* ATCC BAA-1705 was used as a positive control. *K. pneumoniae* ATCC BAA-1706 was used as a negative control.

### 4.5. Plasmid Profiling

Plasmid DNA was extracted from all isolates by the alkaline lysis method using the Zyppy™ Plasmid Miniprep Kit (Zymo Research, Irvine, CA, USA) according to the manufacturer instructions. Seven microliters of each plasmid DNA sample were mixed with 1.5 µL of loading dye (6X), and the mixture was loaded on 0.7% agarose. GeneRuler^TM^ one kilo-base DNA linear Ladder (Fisher Scientific, Loughborough, UK) was used as a reference marker. Samples were electrophoresed for one hour at 150 V in a 1X Tris-borate- EDTA buffer, and the gel was photographed using a UV transilluminator. No restriction enzymes were utilized before electrophoretic separation. Hence, plasmid sizes measured were estimates based on the size of supercoiled plasmid DNA. A clinical isolate of *E. coli* previously identified to possess plasmids was used as a positive control. A clinical isolate previously identified to have no plasmids was used as a negative control.

### 4.6. Total DNA Extraction

Total genomic (chromosomal and plasmid) DNA was extracted from the isolates using a simple boiling method. Briefly, two pure bacterial colonies were inoculated into 5 mL of LB broth and the tubes were incubated overnight at 37 °C for 16 h. Next, 1.5 mL of overnight culture was transferred to an Eppendorf tube and centrifuged at 13,000× *g* for 10 min. The bacterial pellet was suspended in 300 μL sterile water and heated at 100 °C for 10 min to lyse the cells. Debris were removed by centrifugation at 13,000× *g* for 10 min and the supernatant was transferred into an Eppendorf tube. The extracted DNA was stored at −20 °C until used for PCR.

### 4.7. PCR for Isolates’ Identification and Detection of Resistance Genes

PCR was performed to confirm the identity of the 157 isolates by detecting the *E. coli*-specific *uspA*, as described previously [47]. The 25 µL PCR contained 12.5 µL 2X PCR master mix solution, 2 µL template bacteria DNA solution, 1.5 µL of both forward and reverse primers for each of the targeted genes (6.25 pmoles/µL) and 7.5 µL nuclease free water. The following PCR conditions were used; 5 min at 94 °C, followed by 30 cycles of 94 °C for 2 min, 70 °C for 1 min, and 72 °C for 1 min. The 109 isolates confirmed as *E. coli* (*uspA* positive) underwent PCR for detection of virulence and resistance genes. Multiplex PCR was performed to detect *eae* of EPEC, in addition to *aaiC* and *aat* of EAEC, as described previously [19]. Three pairs of primers, were used for amplification of these genes. The 25 µL PCR contained 12.5 µL 2X PCR master mix solution, 3 µL template bacteria DNA solution, 0.4 µL of both forward and reverse primers (6.25 pmoles/µL) for each of *aaiC* and *aat*, 0.44 µL of both forward and reverse primers (6.25 pmoles/µL) for *eae*, and 7.02 µL nuclease free water. The following PCR conditions were used; 4 min at 96 °C, followed by 34 cycles of 95 °C for 20 s, 57 °C for 20 s, and 72 °C for 1 min. Touchdown multiplex PCR was performed to detect *stx1* and *stx2* of EHEC as described previously [19,48]. The 25 µL PCR contained 12.5 µL 2X PCR master mix solution, 3 µL template bacteria DNA solution, 0.5 µL of both forward and reverse primers (6.25 pmoles/µL) of each targeted gene, and 7.5 µL nuclease free water. The following PCR conditions were used; 94 °C for 5 min, followed by 40-cycle each of 94 °C for 30 s, 64  °C for 30 s, and 72  °C for 60 s, for two cycles followed by eight cycles having a 2 °C decrease of annealing temperature after every two cycles. When the annealing temperature of 54 °C was reached at cycle 10, the PCR continued with these cycling parameters followed by a final extension at 72 °C for 10 min. PCR was performed to detect *ipaH* of EIEC as described previously [19]. The 25 µL PCR contained 12.5 µL 2X PCR master mix solution, 2 µL template bacteria DNA solution, 2 µL of both forward and reverse primers for the *ipaH* gene (6.25 pmoles/µL), and 6.5 µL nuclease free water. The following PCR conditions were used 95 °C for 5 min, 30 cycles of 95 °C for 50 s, 55 °C for 1.5 min, and 72 °C for 2 min, with a final extension at 72 °C for 7 min. Table 8 lists PCR primers used.

Multiplex PCR assay was performed to detect carbapenem resistance genes (*bla_KPC_, bla_NDM_*, and *bla_VIM_*), as described previously [49]. The 25 µL PCR contained 12.5 µL 2X PCR master mix solution, 1 µL template bacteria DNA solution, 0.75 µL of both forward and reverse primers (6.25 pmoles/µL) for each of the targeted genes, and seven µL nuclease free water. The following PCR conditions were used; 10 min at 94 °C; 36 cycles of 30 s at 94 °C, 40 s at 52 °C, and 50 s at 72 °C, and final extension at 72 °C for 5 min. PCR was performed to detect ESBL genes (*bla_CTX_*, *bla_TEM_*, and *bla_SHV_*), as described previously [50]. The 25 µL PCR contained 12.5 µL 2X PCR master mix solution, 3 µL template bacteria DNA solution, 0.83 µL of both forward and reverse primers (6.25 pmoles/µL) of each targeted gene, and 7.84 µL nuclease free water. The following PCR conditions were used; 5 min at 94 °C, followed by 35 cycles of 30 s at 94 °C denaturation, 30 s at (50 °C for *bla_CTX_*, 52 °C for *bla_TEM_*, and 56 °C for *bla_SHV_*) for annealing, 30 s (60 s for *bla_SHV_*) at 72 °C for extension, and a final elongation step of 5 min at 72 °C. Controls for PCR were *K. pneumoniae* ATCC BAA-1706 (*bla*_KPC_ and *bla*_NDM_ negative control), *K. pneumoniae* ATCC BAA-1705 (*bla*_KPC_ positive control), *K. pneumoniae* ATCC BAA-2146 (*bla*_NDM_ positive control), *K. pneumoniae* ATCC 700603 (*bla*_SHV_ positive control), and *E. coli* ATCC 35218 (*bla*_TEM_ positive control). Amplification products were detected by electrophoretic separation on 1.5% agarose. For genes having no positive control strains, representative PCR bands were sequenced for verification of amplified genes. Table 8 lists PCR primers used.

### 4.8. Statistical Analysis

The SPSS software version 21 (IBM, Armonk, NY, USA) was used to generate the descriptive analysis of raw data, including generation of all frequency tables and cross tabulations. Fisher’s exact test was used to compare frequency data. Associations between plasmid profiles with resistance genes and phenotypes were investigated using Pearson’s Chi-square test. A *p* value equal to or less than 0.05 was considered statistically significant. Full study data are available online as supplementary material (Table S1). 

## 5. Conclusions

In conclusion, 22% of the *E. coli* isolates recovered from drinking water sources in Jordan were potentially intestinal pathogenic strains, most likely being EAEC. Many isolates harbored beta-lactamase resistance genes, had an MDR phenotype, and an ESBL phenotype. Carbapenemase production was infrequent. None of the isolates had similar plasmid profiles.

## Figures and Tables

**Table 1:** Antimicrobial susceptibility results.

Antimicrobial Agent	Susceptible Number (%)	Intermediate Number (%)	Resistant Number (%)
Amoxicillin/clavulanic acid	80 (73.4)	22 (20.2)	7 (6.4)
Aztreonam	97 (89)	4 (3.7)	8 (7.3)
Cefotaxime	94 (86.2)	1 (0.9)	14 (12.9)
Ceftazidime	98 (89.9)	5 (4.6)	6 (5.5)
Ceftizoxime	97 (89)	2 (1.8)	10 (9.2)
Ceftriaxone	94 (86.2)	1 (0.9)	14 (12.9)
Ampicillin	3 (2.7)	4 (3.7)	102 (93.6)
Ciprofloxacin	91 (83.5)	0	18 (16.5)
Levofloxacin	92 (84.4)	1 (0.9)	16 (14.7)
Nitrofurantoin	107 (98)	1 (0.9)	1 (0.9)
Sulfamethoxazole/trimethoprim	63 (57.8)	1 (0.9)	45 (41.3)
Tigecycline	109 (100)	0	0
Doripenem	109 (100)	0	0
Ertapenem	104 (95.4)	3 (2.8)	2 (1.8)
Imipenem	109 (100)	0	0
Meropenem	109 (100)	0	0

**Table 2:** Isolates’ resistance to antimicrobial agent groups.

Number of Antimicrobial Agent Groups	Number of Resistant Isolates	%
0	2 *	1.8
1	52	47.7
2	17	15.6
3	21	19.3
4	7	6.4
5	5	4.6
6	3	2.8
7	2	1.8

Note: * The two isolates were susceptible to all tested antimicrobial agents.

**Table 3:** Association between extended-spectrum lactamase (ESBL) and carbapenemase phenotypes.

Criteria		Carbapenemase Producer (Number)		*p* Value
		No	Yes	
**ESBL Producer (Number)**	No	49	1	0.562
	Yes	57	2	

**Table 4:** Co-association of resistance genes.

Gene		*bla_KPC_*		*bla_NDM_*		*bla_VIM_*		*bla_TEM_*		*bla_SHV_*		*bla_CTX_*	
		Absent	Present	Absent	Present	Absent	Present	Absent	Present	Absent	Present	Absent	Present
***bla_KPC_***	**Absent**	104	0	93	11	32	72	59	45	103	1	53	51
	**Present**	0	5	4	1	2	3	0	5	5	0	1	4
	***p* Value**	-		0.448		0.499		0.018		0.954		0.187	
***bla_NDM_***	**Absent**	93	4	97	0	26	71	55	42	96	1	47	50
	**Present**	11	1	0	12	8	4	4	8	12	0	7	5
	***p* Value**	0.448		-		0.008		0.110		0.890		0.368	
***bla_VIM_***	**Absent**	32	2	26	8	34	0	19	15	34	0	21	13
	**Present**	72	3	71	4	0	75	40	35	74	1	33	42
	***p* Value**	0.499		0.008		-		0.485		0.688		0.065	
***bla_TEM_***	**Absent**	59	0	55	4	19	40	59	0	58	1	30	29
	**Present**	45	5	42	8	15	35	0	50	50	0	24	26
	***p* Value**	0.018		0.110		0.485		-		0.541		0.459	
***bla_SHV_***	**Absent**	103	5	96	12	34	74	58	50	108	0	54	54
	**Present**	1	0	1	0	0	1	1	0	0	1	0	1
	***p* Value**	0.954		0.890		0.688		0.541		-		0.505	
***bla_CTX_***	**Absent**	53	1	47	7	21	33	30	24	54	0	54	0
	**Present**	51	4	50	5	13	42	29	26	54	1	0	55
	***p* Value**	0.187		0.368		0.065		0.459		0.505		-	

**Table 5:** Association between virulence and resistance genes.

Gene		*eae*		*aaic*		*aat*		*stx1*		*stx2*	*ipah*	
		Absent	Present	Absent	Present	Absent	Present	Absent	Present	Absent	Absent	Present
***bla_KPC_***	**Absent**	101	3	100	4	89	15	104	0	104	102	2
	**Present**	5	0	5	0	5	0	4	1	5	5	0
	***p*** **Value**	0.867		0.827		0.470		0.046		NA	0.910	
***bla_NDM_***	**Absent**	94	3	94	3	82	15	97	0	97	96	1
	**Present**	12	0	11	1	12	0	11	1	12	11	1
	***p*** **Value**	0.702		0.377		0.152		0.110		NA	0.209	
***bla_VIM_***	**Absent**	32	2	34	0	33	1	34	0	34	32	2
	**Present**	74	1	71	4	61	14	74	1	75	75	0
	***p*** **Value**	0.229		0.218		0.021		0.688		NA	0.095	
***bla_TEM_***	**Absent**	58	1	59	0	54	5	59	0	59	59	0
	**Present**	48	2	46	4	40	10	49	1	50	48	2
	***p*** **Value**	0438		0.041		0.072		0.459		NA	0.208	
***bla_SHV_***	**Absent**	105	3	104	4	93	15	107	1	108	106	2
	**Present**	1	0	1	0	1	0	1	0	1	1	0
	***p*** **Value**	0.972		0.963		0.862		0.991		NA	0.982	
***bla_CTX_***	**Absent**	53	1	51	3	48	6	54	0	54	52	2
	**Present**	53	2	54	1	46	9	54	1	55	55	0
	***p*** **Value**	0.507		0.302		0.303		0.505		NA	0.243	

NA: *p* Value could not be computed.

**Table 6:** Association between the number of resistance genes and number of plasmids among the isolates.

Number of Resistance Genes	Number of Plasmids							Total	*p* Value
	0	1	2	3	4	5	6		
0	6	2	2	0	0	0	1	11	0.461
1	11	11	3	0	2	1	0	28	
2	23	13	3	1	1	3	0	44	
3	9	5	4	3	2	0	0	23	
4	1	0	1	0	0	0	0	2	
5	1	0	0	0	0	0	0	1	
Total (%)	51 (46.8)	31 (28.4)	13 (11.9)	4 (3.7)	5 (4.6)	4 (3.7)	1 (0.9)	109 (100)	-

**Table 7:** Association between resistance to antimicrobial agent groups and number of plasmids among the isolates.

Resistance to Antimicrobial Agent Groups	Number of Plasmids							Total	*p* Value
	0	1	2	3	4	5	6		
0	1	0	0	0	0	1	0	2	0.176
1	37	17	3	2	3	1	1	64	
2	9	6	7	2	2	1	0	27	
3	2	4	2	0	0	1	0	9	
4	2	2	0	0	0	0	0	4	
5	0	2	1	0	0	0	0	3	
Total (%)	51 (46.8)	31 (28.4)	13 (11.9)	4 (3.7)	5 (4.6)	4 (3.7)	1 (0.9)	109 (100)	-

**Table 8:** Primers used for PCR.

Gene	Primer	Primer Sequence (5′-3′)	Annealing Temperature (°C)	PCR Product Size (bp)	Reference
*uspA*	uspA-F	CCGATACGCTGCCAATCAGT	64	884	[47]
	uspA-R	ACGCAGACCGTAGGCCAGAT			
*eae*	eae-F	CCCGAATTCGGCACAAGCATAAGC	57	881	[19]
	eae-R	CCCGGATCCGTCTCGCCAGTATTCG			
*aaiC*	aaiC-F	ATTGTCCTCAGGCATTTCAC	57	215	[19]
	aaiC-R	ACGACACCCCTGATAAACAA			
*aat*	aat-F	CTGGCGAAAGACTGTATCAT	57	650	[19]
	aat-R	CAATGTATAGAAATCCGCTGTT			
*stx1*	*stx1*-F	CACAATCAGGCGTCGCCAGCGCACTTGCT	64 *	606	[19]
	*stx1*-R	TGTTGCAGGGATCAGTGGTACGGGGATGC			
*stx2*	*stx2*-F	CCACATCGGTGTCTGTTATTAACCACACC	64 *	372	[19]
	*stx2*-R	GCAGAACTGCTCTGGATGCATCTCTGGTC			
*ipaH*	Shig-F	TGGAAAAACTCAGTGCCTCT	55	424	[19]
	Shig-R	CCAGTCCGTAAATTCATTCT			
*bla_KPC_*	KPC-Fm	CGTCTAGTTCTGCTGTCTTG	52	798	[49]
	KPC-Rm	CTTGTCATCCTTGTTAGGCG			
*bla_NDM_*	NDM-F	GGTTTGGCGATCTGGTTTTC	52	621	[49]
	NDM-R	CGGAATGGCTCATCACGATC			
*bla_VIM_*	VIM-F	GATGGTGTTTGGTCGCATA	52	390	[49]
	VIM-R	CGAATGCGCAGCACCAG			
*bla_TEM_*	TEM-F	ACATGGGGGATCATGTAACT	52	421	[50]
	TEM-R	GACAGTTACAATGCTTACT			
*bla_SHV_*	SHV-F	ATGCGTTATATTCGCCTGTG	56	859	[50]
	SHV-R	AGCGTTGCCAGTGCTCGATG			
*bla_CTX_*	CTX-MU1	ATGTGCAGYACCAGTAARGT	50	593	[50]
	CTX-MU2	TGGGTRAARTARGTSACCAGT			

Notes: * PCR involved 40 cycles having initial annealing at 64 °C for two cycles followed by eight cycles having a 2 °C decrease in annealing temperature after every two cycles.

## Supplementary Materials

The following are available online at https://www.mdpi.com/2076-0817/8/2/86/s1, Table S1: Full data sets in Microsoft Excel file format.

## Appendix A

**Table A1:** Correlation between ESBL phenotype and antimicrobial susceptibility.

Antimicrobial Agent		ESBL Producer		*p* Value
		No	Yes	
		Number (%)	Number (%)	
AMC	Not susceptible	9 (18)	20 (33.9)	0.048
	Susceptible	41 (82)	39 (66.1)	
ATM	Not susceptible	1 (2)	11 (18.6)	0.005
	Susceptible	49 (98)	48 (81.4)	
CTX	Not susceptible	1 (2)	14 (23.7)	0.001
	Susceptible	49 (98)	45 (76.3)	
CAZ	Not susceptible	2 (4)	9 (15.3)	0.049
	Susceptible	48 (96)	50 (84.7)	
ZOX	Not susceptible	1 (2)	11 (18.6)	0.005
	Susceptible	49 (98)	48 (81.4)	
CRO	Not susceptible	1 (2)	14 (23.7)	0.001
	Susceptible	49 (98)	45 (76.3)	
AM	Not susceptible	47 (94)	59 (100)	0.093
	Susceptible	3 (6)	-	
CIP	Not susceptible	10 (20)	8 (13.6)	0.259
	Susceptible	40 (80)	51 (86.4)	
LEV	Not susceptible	10 (20)	7 (11.9)	0.184
	Susceptible	40 (80)	52 (88.1)	
F	Not susceptible	2 (4)	-	0.208
	Susceptible	48 (96)	59 (100)	
SXT	Not susceptible	19 (38)	27 (45.8)	0.267
	Susceptible	31 (62)	32 (54.2)	
TGC	Susceptible	50 (100)	59 (100)	NA
DOR	Susceptible	50 (100)	59 (100)	NA
ETP	Not susceptible	2 (4)	3 (5.1)	0.579
	Susceptible	48 (96)	56 (94.9)	
IPM	Susceptible	50 (100)	59 (100)	NA
MEM	Susceptible	50 (100)	59 (100)	NA

Notes: Percentage is calculated within the column of each antimicrobial agent. NA: *p* value could not be computed. AMC: Amoxicillin/clavulanic acid. ZOX: Ceftizoxime. CAZ: Ceftazidime. CRO: Ceftriaxone. CTX: Cefotaxime. ATM: Aztreonam. AM: Ampicillin. SXT: Sulfamethoxazole/trimethoprim. F: Nitrofurantoin. LEV: Levofloxacin. CIP: Ciprofloxacin. TGC: Tigecycline. IPM: Imipenem. MEM: Meropenem. ETP: Ertapenem. DOR: Doripenem.

## Appendix B

**Table A2:** Correlation between carbapenemase phenotype and antimicrobial susceptibility.

Antimicrobial Agent		Carbapenemase Producer		*p* Value
		No	Yes	
		Number (%)	Number (%)	
AMC	Not susceptible	26 (24.5)	3 (100)	0.017
	Susceptible	80 (75.5)	0	
ATM	Not susceptible	10 (9.4)	2 (66.7)	0.032
	Susceptible	96 (90.6)	1 (33.3)	
CTX	Not susceptible	13 (12.3)	2 (66.7)	0.049
	Susceptible	93 (87.7)	1 (33.3)	
CAZ	Not susceptible	9 (8.5)	2 (66.7)	0.026
	Susceptible	97 (91.5)	1 (33.3)	
ZOX	Not susceptible	10 (9.4)	2 (66.7)	0.032
	Susceptible	96 (90.6)	1 (33.3)	
CRO	Not susceptible	13 (12.3)	2 (66.7)	0.049
	Susceptible	93 (87.7)	1 (33.3)	
AM	Not susceptible	103 (97.2)	3 (100)	0.919
	Susceptible	3 (2.8)	0	
CIP	Not susceptible	16 (15.1)	2 (66.7)	0.070
	Susceptible	90 (84.9)	1 (33.3)	
LEV	Not susceptible	15 (14.2)	2 (66.7)	0.063
	Susceptible	91 (85.8)	1 (33.3)	
F	Not susceptible	2 (1.9)	0	0.945
	Susceptible	104 (98.1)	3 (100)	
SXT	Not susceptible	43 (40.6)	3 (100)	0.072
	Susceptible	63 (59.4)	0	
TGC	Susceptible	106 (100)	3 (100)	NA
DOR	Susceptible	106 (100)	3 (100)	NA
ETP	Not susceptible	5 (4.7)	0	0.867
	Susceptible	101 (95.3)	3 (100)	
IPM	Susceptible	106 (100)	3 (100)	NA
MEM	Susceptible	106 (100)	3 (100)	NA

Notes: Percentage is calculated within the column of each antimicrobial agent. NA: *p* value could not be computed. AMC: Amoxicillin/clavulanic acid. ZOX: Ceftizoxime. CAZ: Ceftazidime. CRO: Ceftriaxone. CTX: Cefotaxime. ATM: Aztreonam. AM: Ampicillin. SXT: Sulfamethoxazole/trimethoprim. F: Nitrofurantoin. LEV: Levofloxacin. CIP: Ciprofloxacin. TGC: Tigecycline. IPM: Imipenem. MEM: Meropenem. ETP: Ertapenem. DOR: Doripenem.

## Appendix C

**Table A3:** Isolates’ plasmid profiles.

Isolate ID	Plasmid Number	Plasmid Size Estimate (bp)
1	0	NA
5	4	4000\3000\2400\1500
8	0	NA
10	0	NA
12	0	NA
13	1	3000
15	1	4000
17	0	NA
18	1	4200
19	3	7000\3200\2800
20	3	2500\1700\1000
21	1	6000
31	6	8000\7000\5000\3500\2550\1600
32	1	1000
33	1	3500
34	2	6500\2000
37	5	4000\2500\1800\1500\1100
38	1	5000
45	0	NA
46	0	NA
48	0	NA
49	1	4000
62	5	6000\5000\3500\1400\1250
63	0	NA
66	0	NA
68	1	1300
70	0	NA
71	0	NA
73	0	NA
77	0	NA
78	0	NA
81	5	8000\4800\1900\1600\1400
83	0	NA
86	1	2800
87	0	NA
88	0	NA
89	0	NA
90	0	NA
93	1	3500
100	1	3000
101	0	NA
103	0	NA
104	0	NA
105	5	8000\4000\3000\1800\1500
107	1	1300
108	1	3950
110	0	NA
113	1	8000
115	1	7000
116	0	NA
117	4	7000\5000\3500\2400
122	0	NA
123	0	NA
124	0	NA
126	1	1400
127	2	2000\1800
129	2	6000\5000
130	1	6000
135	1	2000
136	2	4000\1300
143	2	3000\2400
149	4	4000\2000\1600\1000
150	3	3000\2000\1990
151	2	1900\1500
152	0	NA
157	0	NA
158	0	NA
159	1	1200
161	1	3000
163	2	2900\2100
164	0	NA
169	0	NA
171	1	3000
173	2	8000\6000
174	2	5000\1500
175	0	NA
177	4	5000\4000\2500\1250
179	0	NA
180	1	2800
185	2	6000\2400
186	1	3500
189	1	2000
194	0	NA
195	1	2800
241	0	NA
242	4	4800\4000\3000\2600
245	3	6000\4600\1400
246	1	1800
247	0	NA
248	1	2300
250	0	NA
253	0	NA
255	1	4000
260	0	NA
261	0	NA
262	0	NA
263	0	NA
266	0	NA
267	0	NA
268	0	NA
269	0	NA
271	2	4600\3500
272	0	NA
274	0	NA
275	1	1300
280	2	8000\3500
281	0	NA
282	2	6000\4200
284	1	2000

Note: NA: isolate did not carry any plasmid.
